# Molecular Markers in Melanoma Progression: A Study on the Expression of miRNA Gene Subtypes in Tumoral vs. Benign Nevi

**DOI:** 10.3390/curroncol31050220

**Published:** 2024-05-17

**Authors:** Mihaela Prodan, Sergiu Costescu, Ahmed Elagez, Sorina Maria Denisa Laitin, Vlad Bloanca, Zorin Crainiceanu, Edward Seclaman, Ana-Olivia Toma, Roxana Manuela Fericean, George Puenea, Gabriel Veniamin Cozma

**Affiliations:** 1 Doctoral School, “Victor Babes” University of Medicine and Pharmacy Timisoara, 300041 Timisoara, Romania; mihaela.prodan@umft.ro; 2 Department of Plastic Surgery, “Pius Brinzeu” Timis County Emergency Clinical Hospital, 300723 Timisoara, Romania; 3 Department of Obstetrics and Gynecology, “Victor Babes” University of Medicine and Pharmacy Timisoara, 300041 Timisoara, Romania; costescu.sergiu@umft.ro; 4 Department of Obstetrics and Gynecology, Oravita City Hospital, 325600 Oravita, Romania; 5 Department of General Medicine, Misr University for Science & Technology, Giza 3236101, Egypt; ahmeddmahmouudd@gmail.com; 6 Discipline of Epidemiology, “Victor Babes” University of Medicine and Pharmacy Timisoara, 300041 Timisoara, Romania; 7 Department of Plastic Surgery, “Victor Babes” University of Medicine and Pharmacy Timisoara, 300041 Timisoara, Romania; bloanca.vlad@umft.ro (V.B.); crainiceanu.zorin@umft.ro (Z.C.); 8 Department of Biochemistry and Pharmacology, “Victor Babes” University of Medicine and Pharmacy Timisoara, 300041 Timisoara, Romania; eseclaman@umft.ro; 9 Center for Complex Networks Science, “Victor Babes” University of Medicine and Pharmacy Timisoara, 300041 Timisoara, Romania; 10 Discipline of Dermatology, “Victor Babes” University of Medicine and Pharmacy Timisoara, 300041 Timisoara, Romania; toma.olivia@umft.ro (A.-O.T.); manuela.fericean@umft.ro (R.M.F.); 11 Department of Dermatology, Timisoara Municipal Emergency Hospital, 300254 Timisoara, Romania; 12 Department XVI, “Victor Babes” University of Medicine and Pharmacy Timisoara, 300041 Timisoara, Romania; puenea.george@umft.ro; 13 Department of Surgical Semiology I and Thoracic Surgery, “Victor Babes” University of Medicine and Pharmacy of Timisoara, 300041 Timisoara, Romania; gabriel.cozma@umft.ro

**Keywords:** melanoma, microRNA, immunotherapy, biomarkers, oncology

## Abstract

This study investigates the differential expression of miRNA gene subtypes in tumoral versus benign nevi in individuals with melanoma, aiming to identify clinically significant correlations that could serve as reliable markers for assessing tumor stage and progression. Conducted between 2019 and 2022, this descriptive, quantitative observational research analyzed 90 formalin-fixed paraffin-embedded (FFPE) samples from the Pius Brinzeu County Emergency Clinical Hospital, Timisoara, including 45 samples of advanced-stage melanoma and 45 samples of pigmented nevi. miRNA purification and analysis were performed using the miRNeasy Kit and the Human Cancer PathwayFinder miScript miRNA PCR Array, with statistical analysis (including logistic regression) to determine associations with cancer staging, such as high Breslow index risk, number of mitoses, and vascular invasion. After the analysis and comparison of 180 miRNA gene subtypes, we selected 10 of the most upregulated and 10 most downregulated genes. The results revealed that hsa-miR-133b, hsa-miR-335-5p, hsa-miR-200a-3p, and hsa-miR-885-5p were significantly upregulated in melanoma samples, with fold changes ranging from 1.09 to 1.12. Conversely, hsa-miR-451a and hsa-miR-29b-3p showed notable downregulation in melanoma, with fold changes of 0.90 and 0.92, respectively. Additionally, logistic regression analysis identified hsa-miR-29b-3p (OR = 2.51) and hsa-miR-200a-3p (OR = 2.10) as significantly associated with an increased risk of a high Breslow index, while hsa-miR-127-3p and hsa-miR-451a were associated with a reduced risk. Conclusively, this study underscores the significant alterations in miRNA expression in melanoma compared to benign nevi and highlights the potential of specific miRNAs as biomarkers for melanoma progression. The identification of miRNAs with significant associations to melanoma characteristics suggests their utility in developing non-invasive, cost-effective diagnostic tools and in guiding therapeutic decisions, potentially improving patient outcomes in melanoma management.

## 1. Introduction

Melanoma is the leading cause of death related to skin cancer [1,2,3,4]. Despite the approval of targeted therapies and immunotherapy by the FDA, the survival rate of metastatic melanoma remains low, at around 10–15%. Immunotherapy has shown significant benefits, improving overall survival by approximately 35–50% for melanoma treatment [1,5,6,7]. Early immunotherapies for advanced melanoma included interferon (IFN)-alpha and interleukin-2, but these were associated with severe toxicity and a low rate of long-term complete response [2,3,4,8,9,10]. Subsequent immunotherapies focused on monoclonal antibodies targeting immune checkpoint proteins, with the first checkpoint inhibitors being anti-CTLA-4 agents, ipilimumab, and tremelimumab, although about 50% of melanoma patients either do not respond or develop resistance to immune checkpoint inhibitors [3,11,12].

MicroRNAs (miRNAs) are non-coding RNAs that regulate both transcription and translation, and their abnormal expression is commonly found in cancers, including melanoma. miRNAs play important roles in modulating the biological functions of both tumor and immune cells. Recent studies have highlighted the modulation of miRNAs in innate and adaptive immunity by regulating the differentiation and activation of immune cells. Clinical trials targeting specific miRNAs and miRNA inhibitors, such as anti-miR-21 and anti-miR-155, have been conducted, underscoring the central role of miRNAs in tumor progression and the tumor microenvironment [13,14,15,16].

Advancements in sequencing technologies have positioned non-coding RNAs (ncRNAs) as promising biomarkers for monitoring melanoma progression and recurrence. Several miRNAs have been identified as key regulators in melanoma development and can serve as diagnostic or prognostic markers. Public databases like TCGA provide a platform for exploring the role of specific miRNA expression patterns in melanoma and the development of bioinformatic diagnostic tools. However, single-biomarker-based tools have limitations, and comprehensive multi-omic analyses are necessary for identifying specific and precise prognostic biomarkers in melanoma [6,10,11,12].

The emerging evidence has highlighted the significant contribution of miRNAs to the immune response and therapeutic options in cancer. Understanding the biological roles of miRNAs in tumor immunity is essential. The protein kinase C (PKC) family, involved in both the normal biology of melanocytes and the pathology of melanoma, plays oncogenic and tumor-suppressive roles [17]. Elucidating the diverse and context-dependent functions of PKC enzymes in melanocyte/melanoma biology is key to leveraging these kinases as therapeutic targets. The definitive diagnosis of melanoma is provided by the histopathological examination of tissue samples from lesions. Due to the cutaneous locations where excision can cause significant functional impairment or aesthetic damage, auxiliary diagnostic methods are helpful in the early diagnosis and treatment of melanoma. New genetic sequencing methods aim to detect these molecular-level mutations that play a significant role in melanoma development, providing important diagnostic and prognostic information and aiding in the development of targeted melanoma treatments. Current melanoma diagnosis relies on clinical evaluation, histopathology, and imaging techniques like dermoscopy and MRI, which are essential for staging and progression assessment. Molecular diagnostics are augmenting these methods, particularly through the potential use of miRNAs as biomarkers. miRNAs like miR-21 and miR-155 are linked to melanoma progression and have been highlighted as promising non-invasive biomarkers for assessing tumor behavior and prognosis. These biomarkers could complement traditional diagnostics, enhancing detection and therapeutic targeting in melanoma [18].

This study aims to deepen research on miRNAs in tumor tissue, seeking clinically significant correlations between the expression of miRNA gene variants in benign nevi and malignant melanoma tissues in individuals diagnosed with melanoma, potentially offering a reliable method with high clinical value for assessing tumor stage and progression. This quantitative determination could provide a more accessible and cost-effective diagnostic method than the current standard—surgical excision followed by histopathological examination—and could also help in assessing the success of curative interventions and detecting metastasis through repeat testing at set intervals.

## 2. Materials and Methods

### 2.1. Study Design

Selected patients for the study were diagnosed with advanced-stage melanoma between 2019 and 2022, whose primary tumor lesions had been excised in the Plastic Surgery Department of Pius Brinzeu County Emergency Clinical Hospital, Timisoara, and histopathologically examined in the hospital’s Department of Pathology. 

The study was designed as a descriptive, quantitative observational research project to evaluate patients diagnosed with melanoma. The study distinguished between two subgroups for each patient: one comprising tumor tissue samples and the other comprising benign nevi samples from the same patients diagnosed with melanoma. All participants had provided informed consent, and the study had received ethical approval from the hospital’s Ethical Committee.

The research hypothesis aimed to verify that, as with other carcinomas such as ovarian, esophageal, and colorectal cancers, the progression of the disease correlates with increased expression or downregulation of miRNA gene subtypes in both tumor-infiltrated tissues and benign nevi. 

### 2.2. Sample Collection

A total of 45 malignant melanoma and 45 samples of pigmented nevi were analyzed. These samples consisted of formalin-fixed paraffin-embedded (FFPE) slides obtained from the archive of the Department of Pathology at the Pius Brinzeu County Emergency Clinical Hospital, Timisoara. The FFPE slides included cases of cutaneous melanoma (stages III–IV) and pigmented nevi. To minimize genomic and transcriptional changes that could arise due to environmental factors, the control group was composed of pigmented nevi samples from individuals aged between 18 and 35 years that were age-matched to minimize age-related genomic changes and isolate miRNA expression differences due to melanoma pathology. Patients did not receive any treatment prior to the skin excision from which the tissue sample was obtained. The population in which it is proposed to demonstrate the reproducibility of the results obtained are patients diagnosed with melanoma: sex (male/female), age (between 18 and 90 years), skin subtype of Fitzpatrick classification (I, II, III, IV), and stage of disease (I, II, III, IV).

### 2.3. miRNA Purification and Analysis

miRNA was purified from the FFPE samples using the miRNeasy Kit (Qiagen, Germantown, MD, USA), following the manufacturer’s instructions. The expression of 84 miRNAs was analyzed using the Human Cancer PathwayFinder miScript miRNA PCR Array (Qiagen, Germantown, MD, USA) in a 7900HT real-time PCR instrument (Thermo Fisher Scientific, Waltham, MA, USA).

### 2.4. Data Analysis

Real-time PCR data were analyzed using the online QIAGEN GeneGlobe Data Analysis Center, with a threshold set at 2 for miRNA expression changes in melanoma samples. Setting a threshold of 2 for miRNA expression changes served to identify significant alterations in miRNA levels associated with melanoma, distinguishing them from background noise. This threshold implies that only miRNAs that exhibit at least a twofold increase or decrease in expression are considered relevant, focusing on changes that are more likely to have biological and clinical significance and ensuring that observed differences are statistically and biologically meaningful, reducing the likelihood of false positives due to minor fluctuation in miRNA expression that might not impact melanoma progression or diagnosis. 

Results from the melanoma sample group and the control group consisting of pigmented nevi were compared. Adjustments were made using the Benjamini–Hochberg procedure to eliminate any false discovery rates. Qiagen Ingenuity Pathway Analysis (IPA) was also utilized to identify pathways and pathologies associated with miRNA mutations.

### 2.5. Statistical Analysis

Data analysis was performed using SPSS version 27. A convenience method was considered to calculate the sample size, using a confidence level of 95% for a 5% margin of error, with a necessary minimum of 42 cases. The statistical power was considered to be 80%. Descriptive statistics provided a summary of demographic and clinical characteristics. The proportions were described as *n* (%) and were compared using the Chi-square test or Fisher’s exact test based on the frequency assumptions. For continuous data, we calculated the independent samples t-test when comparing two means. Spearman’s or Pearson’s correlation coefficients were calculated to determine the strength of associations between study variables. Logistic regression was used to identify the influence of high Breslow index risk. A *p*-value < 0.05 was considered statistically significant.

## 3. Results

The current study evaluated various characteristics among 45 patients with malignant melanoma and 45 individuals with benign nevi to identify differences in expression patterns of miRNA gene subtypes in tumoral versus benign nevi. The mean age of individuals with malignant melanoma was 52.4 years (SD = 11.0), compared to 50.2 years (SD = 9.2) for those with benign nevi, demonstrating no statistically significant difference between the two groups (*p*-value = 0.306). Similarly, gender distribution between males (60.0% in malignant melanoma vs. 48.9% in benign nevi) and females (40.0% in malignant melanoma vs. 51.1% in benign nevi) showed no significant differences (*p*-value = 0.289).

Notable differences emerged in the analysis of pigmentation, presence of ulceration, and presence of necrosis. A significantly higher percentage of individuals with malignant melanoma exhibited heavy pigmentation (64.4%) compared to those with benign nevi (26.7%), with a *p*-value of <0.001. This suggests a strong association between heavy pigmentation and the occurrence of malignant melanoma. Furthermore, the presence of ulceration was significantly more common in malignant melanoma (53.3%) than in benign nevi (13.3%), with a *p*-value of <0.001. Similarly, necrosis was observed more frequently in malignant melanoma cases (42.2%) than in benign nevi cases (4.4%), also with a *p*-value of <0.001. These findings indicate that the presence of ulceration and necrosis is associated with more aggressive melanoma characteristics.

Location analysis showed that malignant melanomas were distributed across the occipital region (26.7%), scapular region (22.2%), subscapular region (28.9%), and lumbar region (22.2%). In contrast, benign nevi were found predominantly in the lumbar region (37.8%) and less so in the other regions (occipital 20.0%, scapular 24.4%, subscapular 17.8%). However, the differences in location distribution did not reach statistical significance (*p*-value = 0.323), as presented in Table 1.

Breslow thickness was reported with a mean of 2.0 mm for malignant melanoma cases. The tumor infiltration depth significantly differed between malignant melanoma (mean 2.0 ± 1.1 mm) and benign nevi (mean 0.3 ± 0.1 mm), with a *p*-value of <0.001, indicating a deeper infiltration in melanoma tissues. The distribution of Clark levels was exclusively reported for malignant melanoma: 13.3% at levels I–II, 48.9% at levels III–IV, and 37.8% at level V. The mitotic rate was found to be high in 40% of the melanoma cases. Immunohistochemical markers showed a significant difference in the expression of S100 between malignant melanoma (66.7%) and benign nevi (31.1%), with a *p*-value of <0.001. However, the difference in the expression of HMB45 between malignant melanoma (57.8%) and benign nevi (42.2%) was not statistically significant (*p*-value = 0.140).

The majority of melanoma cases (91.1%) were excised with safety margins. The appearance of tumors was classified as nodular, polypoid, or epithelioid, with no significant difference observed in the distribution of these appearances between melanoma and benign nevi cases (*p*-value = 0.540). The presence of tumor emboli in blood vessels was noted in 15.6% of melanoma cases. The inflammatory response was significantly more common in malignant melanoma (75.6%) than in benign nevi (28.9%), with a *p*-value of <0.001. The immune cell infiltrate type was predominantly lymphocytic in melanoma (64.7%) compared to benign nevi (46.2%), although this difference was not statistically significant (*p*-value = 0.114), as described in Table 2.

The gene hsa-miR-133b showed a mean expression level of 34.82 in malignant melanoma compared to 31.17 in benign nevi, with a fold change of 1.12 and a statistically significant *p*-value of <0.001. Hsa-miR-335-5p’s expression was 34.15 in melanoma against 31.29 in nevi, achieving a fold change of 1.09, also with a *p*-value of <0.001. Hsa-miR-200a-3p was expressed at a mean level of 34.07 in melanoma versus 30.96 in nevi, showing a fold change of 1.10, with its difference being significant (*p*-value < 0.001). Similarly, hsa-miR-885-5p presented a mean expression of 35.31 in melanoma and 32.05 in nevi, with a fold change of 1.10 and a *p*-value of <0.001.

Hsa-miR-20b-5p had a mean expression of 31.87 in melanoma compared to 29.21 in nevi, with a fold change of 1.09 and a *p*-value of <0.001. The gene hsa-miR-7-1-3p exhibited mean expression levels of 34.75 in melanoma and 33.39 in nevi, with a fold change of 1.04 and a *p*-value of 0.014. Hsa-miR-301a-3p showed mean levels of 33.60 in melanoma versus 32.30 in nevi, having a fold change of 1.04 and a *p*-value of 0.016. Hsa-let-7b-3p’s expression was 32.99 in melanoma and 31.63 in nevi, with a fold change of 1.04 and a *p*-value of 0.009. Additionally, hsa-miR-148b-3p and hsa-miR-584-5p were upregulated in melanoma with mean expression levels of 30.78 and 30.94, respectively, compared to 29.17 and 28.73 in benign nevi. Both had fold changes above 1.06 and *p*-values of <0.001, indicating significant upregulation in malignant melanoma, as presented in Table 3.

The data revealed that hsa-miR-451a exhibited a notable downregulation in malignant melanoma with a mean expression level of 31.28, compared to 34.65 in benign nevi, representing a fold change of 0.90 and a highly significant difference with a *p*-value of <0.001. Similarly, hsa-miR-29b-3p was significantly downregulated in melanoma, showing a mean expression level of 31.11 versus 33.93 in nevi, with a fold change of 0.92 and a *p*-value of <0.001. Hsa-miR-361-5p and hsa-miR-495-3p also demonstrated significant downregulation in melanoma, with mean expression levels of 30.25 and 28.19, respectively, compared to higher levels in nevi (32.57 and 30.23), both achieving fold changes of less than 0.94 and *p*-values of <0.001. This trend continued with hsa-miR-590-5p and hsa-miR-127-3p, which showed fold changes of 0.94 and 0.90, respectively, both with *p*-values of <0.001, indicating significant downregulation. Other miRNAs, such as hsa-miR-29a-3p, hsa-miR-18a-5p, and hsa-miR-215-5p, showed modest downregulation with fold changes ranging from 0.95 to 0.96 and *p*-values indicating statistical significance (0.013, 0.003, and 0.002, respectively), pointing towards their potential involvement in melanoma progression through decreased expression (Table 4).

Significant correlations were observed between certain miRNA gene subtypes and the Breslow index. For instance, hsa-miR-335-5p showed a positive correlation with a ρ value of 0.401 and a *p*-value of <0.001, indicating a strong association with melanoma thickness. Similarly, hsa-miR-20b-5p was significantly correlated with the Breslow index (ρ= 0.388, *p*-value = 0.001), and hsa-miR-584-5p also demonstrated a significant correlation (ρ= 0.316, *p*-value < 0.001). Notably, hsa-miR-29b-3p exhibited the strongest correlation with the Breslow index among the miRNAs studied (ρ = 0.565, *p*-value < 0.001).

In terms of mitotic rate, hsa-miR-20b-5p showed a significant positive correlation (ρ = 0.482, *p*-value < 0.001), suggesting its association with higher tumor cell proliferation. hsa-miR-200a-3p also correlated significantly with mitotic rate (ρ = 0.331, *p*-value = 0.006), as did hsa-miR-29b-3p (ρ = 0.279, *p*-value = 0.006). Regarding vascular involvement, hsa-miR-29c-3p showed a positive correlation (ρ = 0.326, *p*-value = 0.008), and hsa-miR-29b-3p also had a significant correlation (ρ = 0.322, *p*-value = 0.046). Additionally, hsa-miR-451a was negatively correlated with vascular involvement (ρ = −0.261, *p*-value = 0.014), suggesting its downregulation might be linked to decreased vascular association in melanoma (Table 5).

The analysis revealed significant associations between several miRNA gene subtypes and the likelihood of having a high Breslow index. hsa-miR-29b-3p emerged as a significant predictor, with a coefficient (β) of 0.916 and an odds ratio (OR) of 2.510, indicating that the presence of this miRNA subtype significantly increases the risk of exhibiting a high Breslow index. Similarly, hsa-miR-200a-3p and hsa-miR-335-5p were also positively associated with a higher risk of a high Breslow index, with ORs of 2.098 and 1.849, respectively, suggesting that increased expression levels of these miRNAs are linked to greater thickness of melanoma lesions.

**Figure 1:**
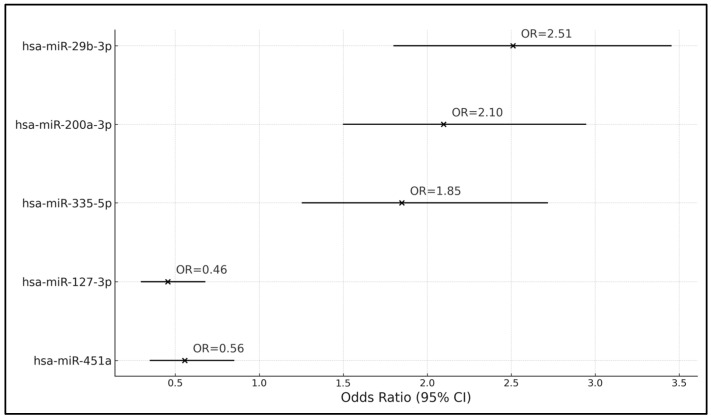
Forest plot of the logistic regression analysis of gene subtypes influencing high Breslow index risk.

On the other hand, hsa-miR-127-3p and hsa-miR-451a were associated with a reduced risk of a high Breslow index. The coefficient for hsa-miR-127-3p was −0.784, translating to an OR of 0.456, indicating a protective effect against a high Breslow index risk. The 95% CI of 0.298–0.675 and a *p*-value of <0.001 further supported this finding. hsa-miR-451a showed a coefficient of −0.587 and an OR of 0.556, with a 95% CI of 0.351–0.848 and a *p*-value of 0.008, similarly indicating a significant decrease in risk associated with its expression (Table 6 and Figure 1).

## 4. Discussion

### 4.1. Literature Findings

The findings from this study indicate that alterations in miRNA expression are associated with melanoma progression. The observed upregulation and downregulation of specific miRNAs in malignant tissues compared to benign nevi are consistent with patterns identified in other types of carcinomas, underscoring the role of miRNAs as both potential oncogenes and tumor suppressors in melanoma. This dual role supports the utility of miRNA profiling in distinguishing between benign and malignant melanocytic lesions, offering a molecular basis for assessing tumor stage and progression.

The findings from this study confirm the initial hypothesis, indicating that alterations in miRNA expression are associated with melanoma progression. The observed upregulation and downregulation of specific miRNAs in malignant tissues compared to benign nevi are consistent with patterns identified in other types of carcinomas, underscoring the role of miRNAs as both potential oncogenes and tumor suppressors in melanoma. Moreover, the risk analysis linking specific miRNA expressions with the likelihood of a high Breslow index further validates the prognostic value of miRNA profiling in melanoma. Given the significant correlations found between miRNA expression levels and clinical-pathological features of melanoma, such as the Breslow index, miRNA profiling emerges as a promising approach for non-invasively assessing tumor characteristics. The capacity to identify specific miRNA signatures associated with tumor aggressiveness and the likelihood of metastasis could refine risk stratification for patients, guiding clinical decision-making towards more personalized treatment plans.

Similarly, Gencia et al. [19] and Chan et al. [20] both contributed to the understanding of miRNA roles in melanoma, although with different emphases and findings. Gencia et al. identified 11 miRNAs with altered expression across melanoma types compared to nevi, including upregulation (e.g., miR-155-5p, miR-9-5p) and downregulation (e.g., miR-205-5p, miR-203a-3p), noting a significant similarity in expression patterns between uveal and mucosal melanomas. Chan et al. delineated miRNA expression in relation to melanoma subtypes and genetic variants, finding seven miRNAs, such as miR-142-3p and miR-486, significantly correlated with acral versus non-acral melanomas (*p* < 0.04) and reported a 25% prevalence of the KRAS-variant in non-acral melanomas, with miR-137 significantly underexpressed in these cases. 

The findings by Rother et al. [21], focusing on the role of tumor suppressors in melanoma progression, and Deacon et al., highlighting the spectrum of genetic and transcriptomic alterations in melanocytic neoplasia, are in accordance with our study’s insights into miRNA expression patterns in melanoma. While Rother et al. detail how the loss of specific tumor suppressors like p16 and RASSF1A contribute to melanoma’s aggressive behavior, our study’s observation of altered miRNA expressions potentially reflects the underlying mechanisms of tumor suppression and oncogenic signaling, akin to the deregulated MAPK signaling pathways. Concurrently, Deacon et al.’s discussion on the clinical potential of melanoma biomarkers echoes our findings’ implications for miRNA-based diagnostics and therapeutics [22]. The emphasis on biomarker validation by Deacon et al. underscores the significance of our study’s miRNAs as candidates for further investigation, aiming to enhance melanoma detection, prognosis, and treatment strategies. These collective findings underscore the complex interplay of genetic and epigenetic factors in melanoma progression, highlighting the utility of miRNA profiling in unveiling melanoma’s molecular intricacies.

The insights presented by Timis et al. on the diagnostic challenges and the molecular underpinnings of malignant melanoma, alongside the therapeutic avenues emerging from the genetic profiling of melanocytes, resonate with our study’s findings on miRNA expression in melanoma [23]. Similarly, Neagu et al.’s review on the differential expression profiles of miRNAs across various skin cancers, including melanoma, and their role in regulating gene expression and tumor progression aligns with our observations [24]. While Timis et al. underscore the complexity of treating advanced-stage melanoma and the continuous search for new therapeutic targets based on genetic insights, our study highlights the potential of miRNAs as such targets, reflecting the genetic and epigenetic alterations driving melanoma. Neagu et al. further elaborate on miRNAs’ role as epigenetic markers, emphasizing their diagnostic and prognostic value, which complements our findings on miRNA profiles as indicators of melanoma’s biological behavior and potential therapeutic targets.

Van Laar et al. [25] and Fawzy et al. [26] both contribute significantly to the field of miRNA research in melanoma with findings that complement and enrich the conclusions drawn from our study. Van Laar et al. discovered 38 circulating miRNAs with significant differences between melanoma patients and normal controls, showcasing the potential of these miRNAs as non-invasive biomarkers for melanoma diagnosis and monitoring, a notion that aligns with our identification of altered miRNA expressions in melanoma tissues. On the other hand, Fawzy et al. focused on the controversial miR-155, uncovering its varied expression in melanoma and its complex associations with disease progression and response to therapy. Their work on miR-155’s dualistic role—both upregulated in early stages and downregulated in advanced melanoma compared to non-cancer tissues—offers a deeper understanding of miRNA functions in melanoma, paralleling our study’s insights into miRNA profiles as indicators of melanoma’s biological behavior and therapeutic targets.

On another note, Reuland et al. [27] identified miR-26a as significantly down-regulated in melanoma cell lines compared to primary melanocytes and demonstrated that treatment with an miR-26a mimic induced apoptosis in melanoma cells by targeting SMAD1 and BAG-4/SODD. This discovery underscores miR-26a’s role as a potential therapeutic target, particularly highlighting the protective role of SODD in melanoma cells against apoptosis. Similarly, Giles et al. [28] investigated miR-7-5p, revealing its tumor-suppressive function in melanoma. They showed that miR-7-5p reduces tumor cell viability, migration, and invasion partly by suppressing NF-κB activity through direct targeting of the RelA subunit. This suppression leads to decreased expression of NF-κB target genes, including pro-inflammatory cytokines. 

The miRNAs, such as miR-21 and miR-155, identified in our study are not specific to melanoma but are also upregulated in various other cancers. miR-21 has been reported in cancers like colorectal, breast, and cervical cancers due to its involvement in crucial regulatory pathways [29,30]. Similarly, miR-155 is implicated in multiple cancers, including breast, lung, and cervical cancers, highlighting its role in cancer-related biological processes [31]. However, miRNAs have multifaceted roles that extend beyond melanoma, such as chronic conditions, as in the case of cystic fibrosis [32,33], or neurodegenerative disease [34,35]. This widespread expression across different cancer types suggests that these miRNAs alone may not serve as specific biomarkers for melanoma without additional, more specific indicators.

There is substantial support in the literature for the role of miRNAs in melanoma, which corroborates our findings of differentially expressed miRNAs in this type of cancer. For instance, studies have highlighted miR-135-a and miR-205, which influence key signaling pathways in melanoma, such as the AKT and NF-κB pathways, impacting tumor behavior and progression. Additionally, miR-146a, which targets genes like NRAS, and miR-155, known to modulate immune responses, are also noted for their roles in melanoma development and progression [36]. Moreover, the comprehensive profiling of miRNA expression in different stages of melanoma, from melanocytes to metastatic melanoma cells, has been shown to provide valuable insights into the role of specific miRNAs. This profiling helps in understanding how miRNAs contribute to tumor growth, angiogenesis, metastasis, drug resistance, and immune responses to cancer [37,38].

The significant correlations between miRNA expression levels and clinical-pathological features like the Breslow index highlight miRNA profiling’s potential in melanoma management. By linking specific miRNAs with tumor depth and aggression, profiling can enhance non-invasive diagnostics and enable precise prognostication. This could lead to more targeted therapeutic strategies, improving personalized treatment plans and potentially enhancing patient outcomes by identifying those at higher risk for aggressive melanoma. 

However, the modest fold changes observed in miRNA expression, though statistically significant, raise questions about their clinical relevance for melanoma diagnostics. In practice, an miRNA biomarker should exhibit not only a statistically significant change but also a fold change substantial enough to influence disease processes. This necessitates setting higher fold change thresholds in clinical settings to ensure that miRNA biomarkers can provide meaningful insights into disease progression and treatment response beyond what is merely detectable statistically.

New miRNA biomarkers identified in our study compared to those previously reported may offer enhanced specificity or sensitivity for melanoma, potentially improving diagnostic accuracy. These differences often arise from variations in study methodologies, population genetics, or stages of melanoma examined. Validation through clinical trials is essential to determine their effectiveness and suitability over established biomarkers, focusing on sensitivity, specificity, and diagnostic value to ascertain their potential utility in clinical settings.

Therefore, this study’s findings underline the necessity for further research to validate miRNA signatures as reliable diagnostic and prognostic tools in clinical settings. Exploring the mechanistic pathways through which these miRNAs influence melanoma progression will be crucial for developing targeted therapeutic strategies. Ultimately, the incorporation of miRNA analysis into clinical practice could enhance the management of melanoma, improving diagnostic precision and tailoring interventions to achieve better patient outcomes. 

### 4.2. Study Limitations

Several limitations are worth mentioning, such as the reliance on formalin-fixed paraffin-embedded (FFPE) samples for miRNA analysis, which could introduce biases due to potential degradation or modification of RNA over time and during processing, potentially affecting the accuracy of miRNA expression levels observed. Additionally, the sample size, while statistically calculated to provide sufficient power, remains relatively small, especially when considering the vast heterogeneity of melanoma in terms of genetic backgrounds, environmental exposures, and disease stages. This diversity could mask subtle variations in miRNA expression that might be significant in specific subtypes or stages of melanoma, limiting the generalizability of the findings. Furthermore, the study’s observational nature restricts the ability to establish causal relationships between miRNA expression patterns and melanoma progression. Future research could benefit from larger, multi-center studies incorporating fresh-frozen samples and longitudinal analyses to validate these findings and elucidate the mechanisms by which specific miRNAs influence melanoma biology and response to therapy.

## 5. Conclusions

In conclusion, the study identifies miRNA expression patterns distinguishing melanoma from benign nevi, suggesting the potential of these miRNAs as biomarkers for melanoma progression. Moreover, these findings pave the way for the development of non-invasive and cost-effective diagnostic methods, offering a promising avenue for enhancing melanoma management and patient care. Through identifying specific miRNAs linked to key melanoma characteristics, this research contributes to the broader effort to refine diagnostic accuracy and therapeutic strategies in melanoma treatment. 

## Figures and Tables

**Table 1:** Sample characteristics of 45 malignant melanoma and 45 benign nevi among clinics in Timisoara, Romania, 2019–2022.

Variables	Malignant Melanoma (*n* = 45)	Benign Nevi (*n* = 45)	*p*-Value
Age (mean ± SD)	52.4 ± 11.0	50.2 ± 9.2	0.306
Gender			0.289
Male	27 (60.0%)	22 (48.9%)	
Female	18 (40.0%)	23 (51.1%)	
Pigmentation			<0.001
Heavy	29 (64.4%)	12 (26.7%)	
Light/None	16 (35.6%)	33 (73.3%)	
Presence of ulceration			<0.001
Yes	24 (53.3%)	6 (13.3%)	
No	21 (46.7%)	39 (86.7%)	
Presence of necrosis			<0.001
Yes	19 (42.2%)	2 (4.4%)	
No	26 (57.8%)	43 (95.6%)	
Location			0.323
Occipital region	12 (26.7%)	9 (20.0%)	
Scapular region	10 (22.2%)	11 (24.4%)	
Subscapular region	13 (28.9%)	8 (17.8%)	
Lumber region	10 (22.2%)	17 (37.8%)	

SD—standard deviation.

**Table 2:** Tissue characteristics from excision of 45 malignant melanoma and 45 benign nevi among clinics in Timisoara, Romania, 2019–2022.

Variables	Malignant Melanoma (*n* = 45)	Benign Nevi (*n* = 45)	*p*-Value
Breslow thickness (mean ± SD, mm)	2.0 ± 1.4	N/A	-
Tumor infiltration depth (mean ± SD, mm)	2.0 ± 1.1	0.3 ± 0.1	<0.001
Clark level			-
I–II	6 (13.3%)	N/A	
III–IV	22 (48.9%)	N/A	
V	17 (37.8%)	N/A	
Mitotic rate			-
High	18 (40%)	N/A	
Low	27 (60%)	N/A	
Immunohistochemical markers (positive)			
S100	30 (66.7%)	14 (31.1%)	<0.001
HMB45	26 (57.8%)	19 (42.2%)	0.140
Resection margins			-
Excised with safety limits	41 (91.1%)	N/A	
Lateral resection margins present	4 (8.9%)	N/A	
Tumor appearance			0.540
Nodular	23 (51.1%)	18 (40.0%)	
Polypoid	12 (26.7%)	16 (35.6%)	
Epithelioid	10 (22.2%)	11 (24.4%)	
Vascular involvement			-
Presence of tumor emboli in blood vessels	7 (15.6%)	N/A	
Absence of tumor emboli	38 (84.4%)	N/A	
Inflammatory response			<0.001
Present	34 (75.6%)	13 (28.9%)	
Absent	11 (24.4%)	32 (71.1%)	
Immune cell infiltrate type			0.114
Lymphocytic	22 (64.7%)	6 (46.2%)	
Plasma cell	8 (23.5%)	2 (15.4%)	
Mixed	4 (11.8%)	5 (38.5%)	

SD—standard deviation.

**Table 3:** Most upregulated miRNA genes in malignant melanoma and benign nevi in Timisoara, Romania, 2019–2022.

Gene	Fold Change (M/C)	Malignant Melanoma (*n* = 45)	Benign Nevi (*n* = 45)	*p*-Value
hsa-miR-133b	1.12	34.82 ± 2.81	31.17 ± 2.64	<0.001
hsa-miR-335-5p	1.09	34.15 ± 2.03	31.29 ± 3.15	<0.001
hsa-miR-200a-3p	1.10	34.07 ± 1.99	30.96 ± 2.61	<0.001
hsa-miR-885-5p	1.10	35.31 ± 2.64	32.05 ± 2.22	<0.001
hsa-miR-20b-5p	1.09	31.87 ± 2.06	29.21 ± 2.43	<0.001
hsa-miR-7-1-3p	1.04	34.75 ± 2.87	33.39 ± 2.29	0.014
hsa-miR-301a-3p	1.04	33.60 ± 2.43	32.30 ± 2.61	0.016
hsa-let-7b-3p	1.04	32.99 ± 2.71	31.63 ± 2.14	0.009
hsa-miR-148b-3p	1.06	30.78 ± 2.02	29.17 ± 2.29	<0.001
hsa-miR-584-5p	1.07	30.94 ± 2.97	28.73 ± 2.31	<0.001

**Table 4:** Most downregulated miRNA genes in malignant melanoma and benign nevi in Timisoara, Romania, 2019–2022.

Gene	Fold Change (M/C)	Malignant Melanoma (*n* = 45)	Benign Nevi (*n* = 45)	*p*-Value
hsa-miR-451a	0.90	31.28 ± 2.06	34.65 ± 2.27	<0.001
hsa-miR-29c-3p	0.97	31.21 ± 2.78	32.28 ± 2.95	0.080
hsa-miR-29b-3p	0.92	31.11 ± 2.94	33.93 ± 2.97	<0.001
hsa-miR-29a-3p	0.95	29.48 ± 2.89	31.00 ± 2.81	0.013
hsa-miR-361-5p	0.93	30.25 ± 2.61	32.57 ± 2.39	<0.001
hsa-miR-18a-5p	0.95	31.34 ± 2.92	32.96 ± 2.10	0.003
hsa-miR-495-3p	0.93	28.19 ± 2.09	30.23 ± 2.68	<0.001
hsa-miR-590-5p	0.94	30.94 ± 2.20	33.04 ± 2.44	<0.001
hsa-miR-215-5p	0.96	29.48 ± 2.05	30.85 ± 2.12	0.002
hsa-miR-127-3p	0.90	27.39 ± 2.33	30.33 ± 2.54	<0.001

**Table 5:** Correlation matrix for miRNA gene subtypes with Breslow index and vascular involvement in samples identified from Timisoara, Romania, 2019–2022.

miRNA Gene Subtype	Breslow Index (ρ)	*p*-Value	Mitotic Rate (ρ)	*p*-Value	Vascular Involvement (ρ)	*p*-Value
hsa-miR-133b	0.351	0.036	0.256	0.059	0.126	0.182
hsa-miR-335-5p	0.401	<0.001	0.313	0.014	0.247	0.052
hsa-miR-200a-3p	0.464	0.015	0.331	0.006	−0.038	0.616
hsa-miR-885-5p	0.197	0.418	0.219	0.033	0.173	0.225
hsa-miR-20b-5p	0.388	0.001	0.482	<0.001	0.278	0.045
hsa-miR-7-1-3p	−0.214	0.099	0.025	0.427	−0.350	0.212
hsa-miR-301a-3p	−0.384	0.001	−0.377	0.007	0.059	0.621
hsa-let-7b-3p	0.132	0.326	−0.170	0.149	0.275	0.038
hsa-miR-148b-3p	0.202	0.080	−0.026	0.713	0.074	0.411
hsa-miR-584-5p	0.316	<0.001	0.198	0.047	0.056	0.304
hsa-miR-451a	−0.359	0.005	−0.139	0.306	−0.261	0.014
hsa-miR-29c-3p	0.240	0.059	0.150	0.081	0.326	0.008
hsa-miR-29b-3p	0.565	<0.001	0.279	0.006	0.322	0.046
hsa-miR-29a-3p	−0.173	0.147	0.079	0.341	0.129	0.094
hsa-miR-361-5p	−0.236	0.248	0.196	0.035	0.242	0.138
hsa-miR-18a-5p	−0.333	0.040	0.244	0.036	−0.012	0.514
hsa-miR-495-3p	0.192	0.715	−0.023	0.439	0.045	0.447
hsa-miR-590-5p	0.050	0.614	0.108	0.054	−0.145	0.189
hsa-miR-215-5p	−0.136	0.134	−0.091	0.318	−0.249	0.045
hsa-miR-127-3p	−0.418	<0.001	−0.349	0.006	−0.384	0.027

**Table 6:** Logistic regression analysis of gene subtypes influencing high Breslow index risk in samples analyzed from Timisoara, Romania, in the period 2019–2022.

Gene Type	Coefficient (β)	SE	OR	95% CI	*p*-Value
hsa-miR-29b-3p	0.916	0.213	2.510	1.803–3.451	<0.001
hsa-miR-200a-3p	0.741	0.189	2.098	1.501–2.943	0.003
hsa-miR-335-5p	0.615	0.221	1.849	1.256–2.715	0.002
hsa-miR-127-3p	−0.784	0.245	0.456	0.298–0.675	<0.001
hsa-miR-451a	−0.587	0.220	0.556	0.351–0.848	0.008

OR—odds ratio; CI—confidence interval; SE—standard error.

## Data Availability

Data available on request.
